# Reserve of global constructive work for early diagnosis of myocardial ischemia and risk stratification in chronic coronary syndrome

**DOI:** 10.3389/fcvm.2025.1598453

**Published:** 2025-08-01

**Authors:** Ruohan Zhao, Jing Zhang, Yu Xie, Yuting Tan, Benling Qi, Lijuan Bai, Jingjing Wu, Min Cheng, Xiang Wang, Qing Lv, Jing Wang, Mingxing Xie

**Affiliations:** ^1^ Department of Ultrasound Medicine, Union Hospital, Tongji Medical College, Huazhong University of Science and Technology, Wuhan, Hubei, China; ^2^ Hubei Province Clinical Research Center for Medical Imaging, Wuhan, China; ^3^ Hubei Province Key Laboratory of Molecular Imaging, Wuhan, China; ^4^ Department of Geriatrics, Union Hospital, Tongji Medical College, Huazhong University of Science and Technology, Wuhan, Hubei, China; ^5^ Department of Cardiology, Union Hospital, Tongji Medical College, Huazhong University of Science and Technology, Wuhan, Hubei, China

**Keywords:** chronic coronary syndrome, myocardial ischemia, myocardial work, stress echocardiography, coronary flow velocity reserve

## Abstract

**Background:**

In chronic coronary syndrome (CCS), assessing myocardial ischemia is difficult due to its variable severity. Myocardial mechanical parameters are helpful in ischemia detection. This study investigates the use of non-invasive myocardial work (MW) for ischemia detection and risk assessment in CCS patients.

**Method:**

The study included 115 patients (70 men, mean age 61 years) with suspected or diagnosed CCS in the derivation cohort and 62 patients in the validation cohort. All patients underwent regadenoson stress echocardiography, with early ischemia indicated by coronary flow velocity reserve (CFVR) <2.5. The patients were categorized based on CFVR, and logistic regression was used to assess the association between myocardial work (MW) and ischemia. Model performance was evaluated for accuracy, prediction, and practicality. The risk stratification thresholds were set by sensitivity and specificity.

**Results:**

Of the 115 patients, 48 (41.74%) had myocardial ischemia. MW was more sensitive in detecting ischemia than global longitudinal strain. Multivariate analysis showed that global constructive work reserve (△GCW) was independently correlated with CFVR, with the highest AUC (0.777). A model including △GCW and hemoglobin identified ischemia with a C-index of 0.844 in the derivation cohort and 0.82 in the validation cohort, allowing calculation of the probability of ischemia in CCS. Risk levels were defined by probabilities of 20% (low) and 70% (high).

**Conclusion:**

The incorporation of △GCW and hemoglobin into the prediction model enhances its ability to estimate myocardial ischemia risk. △GCW offered higher sensitivity and incremental diagnostic value in detecting myocardial ischemia in the heterogeneous CCS population.

## Graphical Abstract

**Figure:**
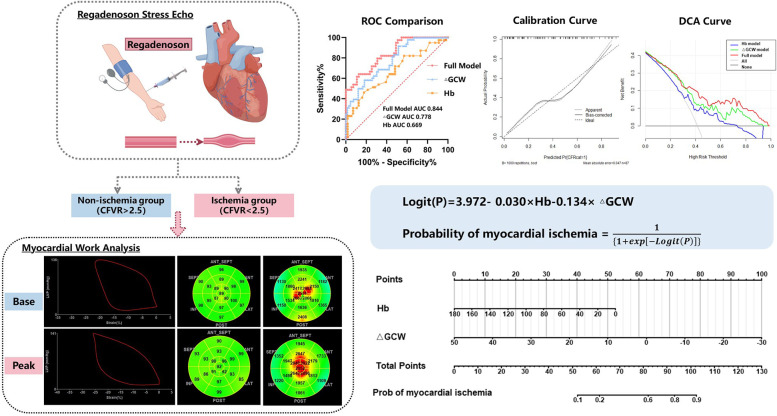

## 1 Introduction

Chronic coronary syndromes (CCS) encompass a range of coronary issues such as microvascular dysfunction and vessel stenosis, leading to significant clinical diversity (1, 2). Current CCS guidelines emphasize myocardial ischemia as a critical factor in decision-making and prognosis assessment (3–7). However, due to the pathophysiological diversity of coronary lesions in CCS, the extent of myocardial ischemia in patients with CCS is highly heterogeneous and complex. Early and accurate identification of myocardial ischemia is challenging in the evaluation of CCS.

Reduced coronary flow velocity reserve (CFVR) is an early indicator of ischemia in both obstructive and non-obstructive CCS (8). The CFVR acquisition rate is lower in exercise/dobutamine SE and in the unskilled compared with vasodilator SE and in the skilled (9, 10).

Myocardial ischemia would induce myocardial mechanical alterations. However, the presentation of myocardial mechanical alteration in different extents of myocardial ischemia may vary. The positivity of regional wall motion abnormality (RWMA) is now declining in SE (11, 12). Thus, it presents a challenge in screening for the most sensitive index of myocardial mechanics in the context of myocardial ischemia. Myocardial work (MW) is a novel index of myocardial mechanics derived from a proprietary left ventricular pressure–strain loop (LV PSL) (13). In comparison to global longitudinal strain (GLS), MW is a superior option in SE. The latter incorporates aspects such as afterload, energy metabolism, and multiparameter analysis, which contribute to its superiority (14, 15). Thus, our study aimed to investigate whether MW was suitable to be applied in the identification of early myocardial ischemia in the context of heterogenous CCS.

## 2 Method

### 2.1 Study population

#### 2.1.1 Derivation cohort

The study prospectively enrolled patients suspected of or diagnosed with CCS in Wuhan Union Hospital, Tongji Medical College, Huazhong University of Science and Technology from January 2022 to December 2023. Other inclusion criteria included successful acquisition of mid-distal left anterior descending coronary (LAD) blood flow and Doppler spectrum; apical four-chamber, three-chamber, and two-chamber views; and age over 18 years. The exclusion criteria were as follows: (1) left ventricular ejection fraction (LVEF) ≤ 50%, significant valvular disease, congenital heart disease, and inherited or acquired cardiomyopathy; (2) patients with contradictions to regadenoson—second-degree/third-degree atrioventricular block, sick sinus syndrome, acute coronary syndrome, decompensated heart failure, excessive low blood pressure, asthma, chronic obstructive pulmonary disease; (3) inadequate acoustic window; and (4) significant cardiac arrhythmia.

All patients underwent transthoracic echocardiography (TTE), including speckle-tracking analysis imaging, MW analysis, and regadenoson SE with an assessment of coronary flow velocity reserve (CFVR) of mid-distal LAD. Myocardial ischemia was defined as CFVR < 2.5 (16). The patients were divided into two groups based on CFVR. All the patients were processed to either coronary angiography or coronary CT angiography after completion of regadenoson SE. The trial was conducted in accordance with the Declaration of Helsinki (as revised in 2013) and was approved by the Institutional Ethics Board of Wuhan Union Hospital, Tongji Medical College, Huazhong University of Science and Technology. All patients provided written informed consent.

#### 2.1.2 Validation cohort

**Figure 1:**
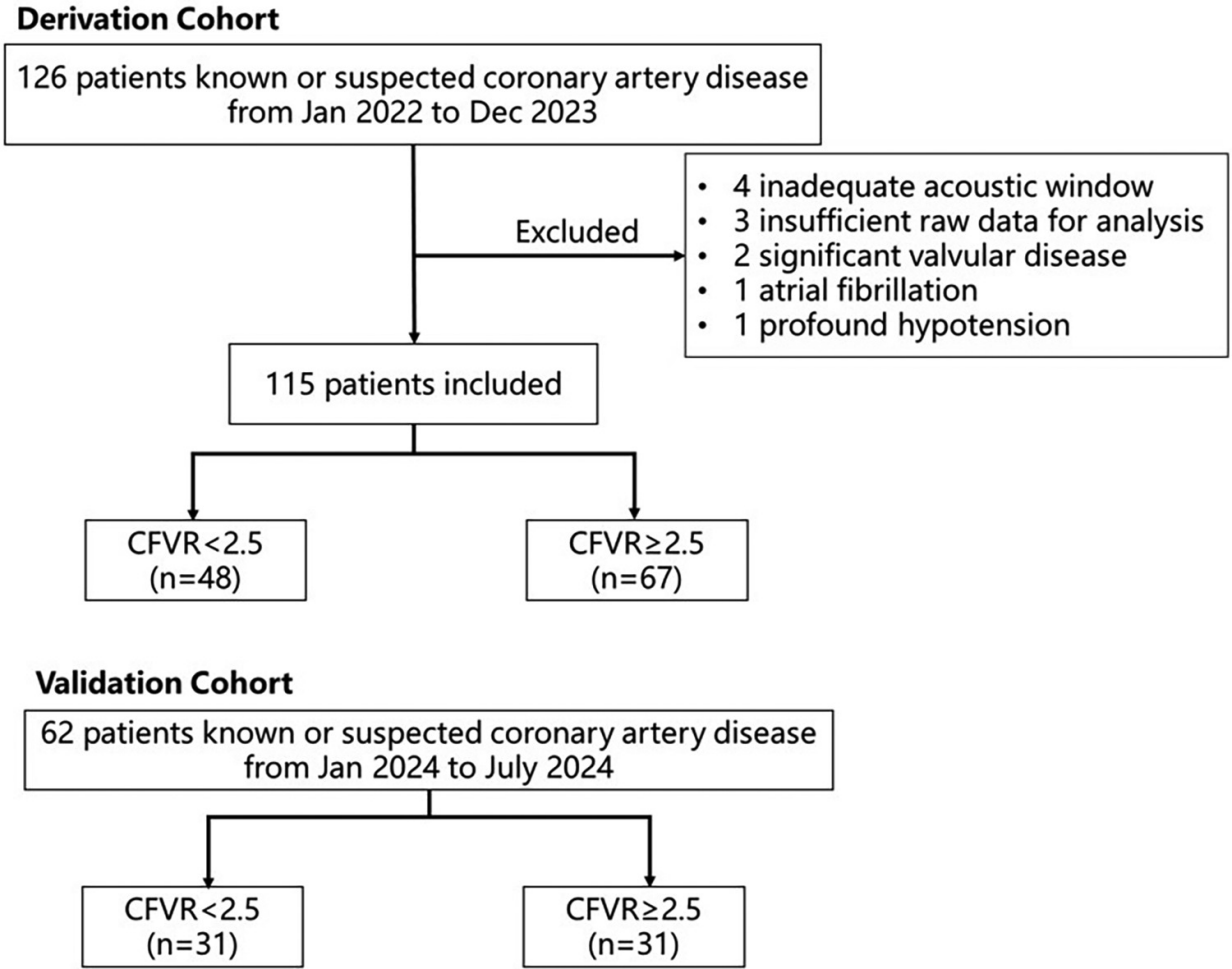
Patient flowchart in the study.

The validation cohort consisted of prospectively enrolled patients who were suspected to have CCS from January 2024 to July 2024. During this period, 62 subjects who met the inclusion and exclusion criteria mentioned above were included in the final analysis to validate the ischemia model (Figure 1).

### 2.2 Conventional echocardiography

Comprehensive conventional TTE at rest was performed using a commercially available ultrasound machine (Vivid E95, GE HealthCare, Horten, Norway), based on the latest guideline (17). Left ventricular ejection fraction (LVEF) was calculated by the biplane Simpson's method. The left atrial volume index (LAVI) was calculated as left atrial volume divided by body surface area. In the apical four-chamber view, Doppler ultrasound was applied to measure mitral valve inflow. Accordingly, mitral valve septal annulus movement was recorded by tissue Doppler imaging.

### 2.3 Regadenoson stress echocardiography and CFVR

Baseline two-dimensional grayscale images were initially recorded from the apical four-chamber, three-chamber, and two-chamber views at frame rates ranging from 50 to 80 fps to facilitate speckle-tracking and myocardial work analysis. We then used Doppler mapping with a 0.25 m/s velocity scale to find the baseline LAD. The scale was actively modified to provide optimal images. The mid-distal LAD was searched in modified apical two- and three-chamber views or modified parasternal short- and long-axis views. A sample volume (1.5–2 mm) was placed on the color signal in the mid-distal LAD to obtain pulse-wave Doppler flowmetry. Finally, the patients underwent regadenoson SE under a dose of 0.4 mg bolus injection according to the latest guideline (18). Pulse-wave Doppler ﬂowmetry of mid-distal LAD and cine loop of apical four-chamber, three-chamber, and two-chamber views at hyperemic peak were recorded for CFVR analysis or stress myocardial work analysis. The interruption criteria were severe, intolerable chest pain, intolerable dyspnea, marked electrocardiography positivity, significant arrhythmia, excessive hypotension [systolic blood pressure (SBP) ≤ 90 mmHg, diastolic blood pressure (DBP) ≤ 60 mmHg], or hypertension (SBP ≥ 220 mmHg, DBP ≥ 120 mmHg). Blood pressure was recorded at baseline and 1 min intervals after regadenoson injection. CFVR was defined as the ratio between hyperemic peak and baseline diastolic coronary flow velocities. CFVR < 2.5 was defined as ischemia. The examination was performed under continuous electrocardiography and blood pressure monitoring. The aminophylline was prepared to reverse regadenoson in necessity.

### 2.4 Speckle-tracking analysis and myocardial work analysis

Left ventricular global longitudinal strain (GLS) was analyzed on a vendor-specific workstation (Echopac version 204; GE Vingmed Ultrasound AS, GE Medical Systems). Following the initiation of the Q-analysis module and manual adjustment of the LV endocardium, the workstation tracked the LV endocardium automatically. The GLS was calculated from the average longitudinal strain of all the LV segments.

MW was calculated on the same workstation. In the dynamic video of the apical three-chamber view, the first frame of the opening and closure of the aortic and mitral valves was selected as the time point of valve switching. After calculating the strain, inputting the stored branchial blood pressure, and identifying the opening and closure of the mitral valve and aortic valve, we could obtain the non-invasive LV pressure–strain loop (LV PSL). Global work index (GWI) is the total work done by the ventricle during mechanical systole (area within the LV PSL curve). Global contractive work (GCW) is positive work performed by a segment in systole and negative work (segment lengthening) during isovolumic relaxation. Global waste work (GWW) is negative work (segment lengthening) during systole and positive work (segment shortening) during isovolumic relaxation. Global work efficiency (GWE) is equal to GCW/(GCW + GWW).

GLS and MW were measured both at baseline and at hyperemic peak. The reserve of LVEF, GLS, or MW is defined as the difference between the peak state and baseline state divided by the baseline state, recorded as Δ.

### 2.5 Coronary angiography or coronary CT angiography

All the patients underwent either coronary angiography or coronary CT angiography. The interval between coronary angiography/coronary CT and SE should be no more than 3 months. Obstructive coronary artery disease (CAD) was defined as ≥50% stenosis in one or more major epicardial vessels.

### 2.6 Statistical analysis

The statistical analysis was performed by SPSS version 25.0 (IBM Corp., Armonk, NY, USA), Medcalc 18.2.1 (MedCalc Software, Ltd., Ostend, Belgium), GraphPad Prism 8.0 (GraphPad Software, Boston, MA, USA), and R version 4.2.3 (R Foundation for Statistical Computing, Vienna, Austria). According to a normal distribution, continuous variables were presented as mean ± SD or median (Q1, Q3). Categorical variables were expressed as number (%). Accordingly, continuous variables were compared either by Student's *t*-test or Mann–Whitney *U* test. Parameters of pre- and post-stress were compared by paired rank sum tests or *t*-tests. The categorical data were analyzed by chi-squared tests or Fisher's exact tests. To avoid problems of overfitting and collinearity, multicollinearity was assessed using collinearity diagnostics (i.e., variance inflation factor >10). The correlation between continuous variables was tested using Spearman's or Pearson's correlation. The independent correlation with CFVR was tested with multivariate stepwise logistic regression. The diagnostic performance of the model and variables was reflected by the receiver operating characteristic (ROC) curve and the area under the curve (AUC). The calibration of the model was reflected by the calibration curve. The model was validated in the validation cohort. All tests were two-sided, and *P* < 0.05 was statistically significant.

## 3 Results

### 3.1 Demographic data and clinical data

The study prospectively included 126 patients with known or suspected coronary artery disease. Eleven patients were excluded (four with inadequate acoustic window, three with insufficient raw data for analysis, two with significant valvular disease, one with atrial fibrillation, one with extensive hypotension), and 115 patients were included in the study. There were 48 patients with CFVR < 2.5, accounting for 41.74% of the study (Figure 1). All the demographic data, clinical characteristics, coronary status, laboratory results, and current medication treatment were compared between the two groups in Table 1. The average age of the enrolled population was 61.00 (56–66.5) years; 61.40% of the subjects were male. As shown in Table 1, there are 64 (55.65%) patients with obstructive CAD. Of these patients, 25% had coronary stenosis between 50% and 70%, and 35.4% had coronary stenosis of 70% or more. Approximately 55.65% of patients' culprit vessel was LAD. There were no significant differences between the two groups in the culprit vessels, stenosis rate, and the number of coronary arteries involved. Patients with CFVR < 2.5 tended to be older (*P* = 0.016) and have lower hemoglobin (*P* = 0.005) than those in patients with CFVR > 2.5. In addition, the baseline characteristics of the two groups were not statistically different.

**Table 1:** Clinical characteristics of the patients.

Variable	Total (*n* = 115)	CFVR ≥ 2.5	CFVR < 2.5	*P*
		(*n* = 67)	(*n* = 48)	
Gender/male, *n* (%)	70 (61.4)	40 (59.7)	30 (62.5)	0.656
Age/year, M (Q₁, Q₃)	61.00 (56, 66.5)	60.00 (54, 65)	63.50 (58.5, 70.25)	0.016
Height/cm, M (Q₁, Q₃)	166.00 (160, 170.5)	165.50 (158, 170)	167.00 (161, 173)	0.158
Weight/kg, M (Q₁, Q₃)	68.00 (60, 74.25)	67.27 (60.25, 73.75)	68.00 (60, 74.50)	0.973
BMI/kg/m^2^, M (Q₁, Q₃)	24.39 (22.31, 26.50)	24.75 (22.5, 26.93)	23.53 (22.04, 25.06)	0.175
HR/bpm, Mean ± SD	71.27 ± 10.83	70.22 ± 10.42	72.73 ± 11.32	0.223
SBP/mmHg, Mean ± SD	129.97 ± 12.26	128.45 ± 11.63	132.08 ± 12.91	0.117
DBP/ mmHg, M (Q₁, Q₃)	80.00 (74.00, 87.5)	80.00 (74, 86)	79.00 (73.75, 89.5)	0.952
HBP, *n* (%)	59 (51.3)	33 (49.25)	26 (54.17)	0.603
DM, *n* (%)	27 (23.68)	17 (25.37)	10 (21.28)	0.613
CCS score				0.135
I	87 (75.65)	54 (80.60)	33 (68.75)	
II	26 (22.61)	13 (19.40)	13 (27.08)	
III	2 (1.74)	0 (0)	2 (4.17)	
IV	0 (0)	0 (0)	0 (0)	
Coronary status				
Obstructive CAD, *n* (%)	64 (55.65)	35 (52.24)	29 (60.42)	0.384
Culprit vessel, *n* (%)				0.700
Non	23 (20.00)	12 (17.91)	11 (22.92)	
LM	1 (0.87)	0 (0)	1 (2.08)	
LAD	64 (55.65)	39 (58.21)	25 (52.08)	
RCA	14 (12.17)	8 (11.94)	6 (12.5)	
LCX	13 (11.3)	8 (11.94)	5 (10.42)	
Vessel involved, *n* (%)				0.832
Single vessel	32 (27.83)	17 (25.37)	15 (31.25)	
Two-vessel	19 (16.52)	11 (16.42)	8 (16.67)	
Three-vessel	13 (11.3)	7 (10.45)	6 (12.5)	
Stenosis rate, %	50 (20,7)	50 (20,7)	50 (14,77.5)	0.587
Stenosis rate classification				0.520
0%–50%	52 (45.2)	33 (49.3)	19 (39.6)	
50%–70%	24 (20.9)	12 (17.9)	12 (25.0)	
70%–100%	39 (33.9)	22 (32.8)	17 (35.4)	
Gensini score	12.75 (3.12,26)	12 (3.12,25.75)	13 (3.12,29.25)	0.543
MI, *n* (%)	8 (6.96)	3 (4.48)	5 (10.42)	0.388
History of PCI, *n* (%)	15 (13.04)	11 (16.42)	4 (8.33)	0.204
Laboratory results				
Hb/g/L, M (Q₁, Q₃)	130.00 (122, 144)	139.00 (125, 146)	125.00 (112, 140)	0.005
Fglu/mmol/L, M (Q₁, Q₃)	5.30 (4.8, 6.1)	5.20 (4.65, 6.05)	5.45 (4.9, 6.2)	0.139
TC/mmol/L, M (Q₁, Q₃)	3.69 (3.18, 4.58)	3.74 (3.38, 4.54)	3.66 (3.13, 4.64)	0.267
TG/mmol/L, M (Q₁, Q₃)	1.17 (0.92, 1.72)	1.29 (0.96, 1.78)	1.09 (0.77, 1.46)	0.128
HDL-c/mmol/L, M (Q₁, Q₃)	1.09 (0.88, 1.37)	1.12 (0.88, 1.3)	1.05 (0.88, 1.39)	0.906
LDL-c/mmol/L, M (Q₁, Q₃)	1.94 (1.53, 2.87)	2.01 (1.53, 2.98)	1.93 (1.53, 2.55)	0.497
NT-proBNP/pg/L, M (Q₁, Q₃)	72.50 (32.5, 104)	73.10 (48.25, 107.75)	65.00 (32.70, 77.8)	0.459
cTNI/ng/L, M (Q₁, Q₃)	2.90 (1.8, 4.5)	2.80 (1.63, 4.27)	3.00 (2.25, 7.6)	0.138
HsCRP/mg/L, M (Q₁, Q₃)	1.20 (0.46, 3.41)	1.24 (0.48, 3.41)	1.13 (0.44, 3.24)	0.814
Medications				
ACEI/ARB, *n* (%)	42 (36.84)	24 (36.36)	18 (37.5)	0.901
Antiplatelet, *n* (%)	73 (64.04)	40 (60.61)	33 (68.75)	0.371
β-blocker, *n* (%)	46 (40.35)	23 (34.85)	23 (47.92)	0.16
Calcium channel blocker, *n* (%)	30 (26.32)	20 (30.3)	10 (20.83)	0.257
Statin, *n* (%)	71 (62.28)	39 (59.09)	32 (66.67)	0.41
Nicorandil, *n* (%)	29 (25.44)	17 (25.76)	12 (25)	0.927

BMI, body mass index; HR, heart rate; SBP, systolic blood pressure; DBP, diastolic blood pressure; HBP, hypertension; DM, diabetes mellitus; CAD, coronary artery disease; LM, left main artery; LAD, left anterior descending artery; RCA, right coronary artery; LCX, left circumflex artery; MI, myocardial infarction; PCI, percutaneous coronary intervention; Hb, hemoglobin; Fglu, fasting glucose; TC, total cholesterol; TG, triglyceride; HDL-c, high density lipoprotein; LDL-c, low density lipoprotein; NT-proBNP, N-terminal B-type natriuretic peptide; cTNI, cardiac troponin I; HsCRP, high-sensitivity C-reactive protein; ACEI, angiotensin-converting enzyme inhibitor; ARB, Angiotensin II Receptor Blocker.

### 3.2 Conventional echocardiographic data

The cardiac chamber quantification, left ventricular systolic function, and diastolic function were compared between the two groups, shown in Table 2. There was no statistical difference in any of the conventional echocardiographic parameters between the two groups.

**Table 2:** Conventional echocardiographic parameters in the two groups.

Variable	Total (*n* = 115)	CFVR ≥ 2.5	CFVR < 2.5	*P*
		(*n* = 67)	(*n* = 48)	
LAVI/ml/m^2^, M (Q₁, Q₃)	23.08 (18.18, 26.62)	21.90 (18.08, 26.03)	24.07 (19.05, 27.3)	0.523
LVEDVI/ml/m^2^, Mean ± SD	41.51 ± 11.83	43.1 ± 13.2	39.55 ± 9.64	0.135
LVESVI/ml/m^2^, M (Q₁, Q₃)	14.93 ± 5.30	15.79 ± 5.31	13.81 ± 5.14	0.060
IVSd/cm, M (Q₁, Q₃)	1.00 (0.9, 1.1)	1.00 (0.9, 1.1)	1.00 (0.9, 1.02)	0.323
RA/cm, M (Q₁, Q₃)	3.40 (3.05, 3.65)	3.30 (3, 3.6)	3.40 (3.1, 3.7)	0.272
RV/cm, Mean ± SD	3.21 ± 0.44	3.18 ± 0.43	3.26 ± 0.44	0.39
LVEF/%, Mean ± SD	65.09 ± 6.25	64.72 ± 5.92	65.61 ± 6.71	0.45
E/A	0.91 ± 0.32	0.92 ± 0.33	0.89 ± 0.32	0.721
E/e′	11.09 ± 3.63	10.91 ± 3.75	11.34 ± 3.47	0.532

LAVI, left atrial volume index; RA, right atrial transversal diameter; RV, right ventricular transversal diameter; LVEDVI, left ventricular end-diastolic volume index; LVESVI, left ventricular end-systolic volume index; IVSd, interventricular septal end-diastolic diameter; RA, right atrium; RV, right ventricle; LVEF, left ventricular ejection fraction; E, E velocity of mitral valve; A, A velocity of mitral valve; e′, early diastolic mitral annular tissue velocity.

### 3.3 Stress echocardiography and myocardial work analysis

#### 3.3.1 Characterizing the response to regadenoson

**Figure 2:**
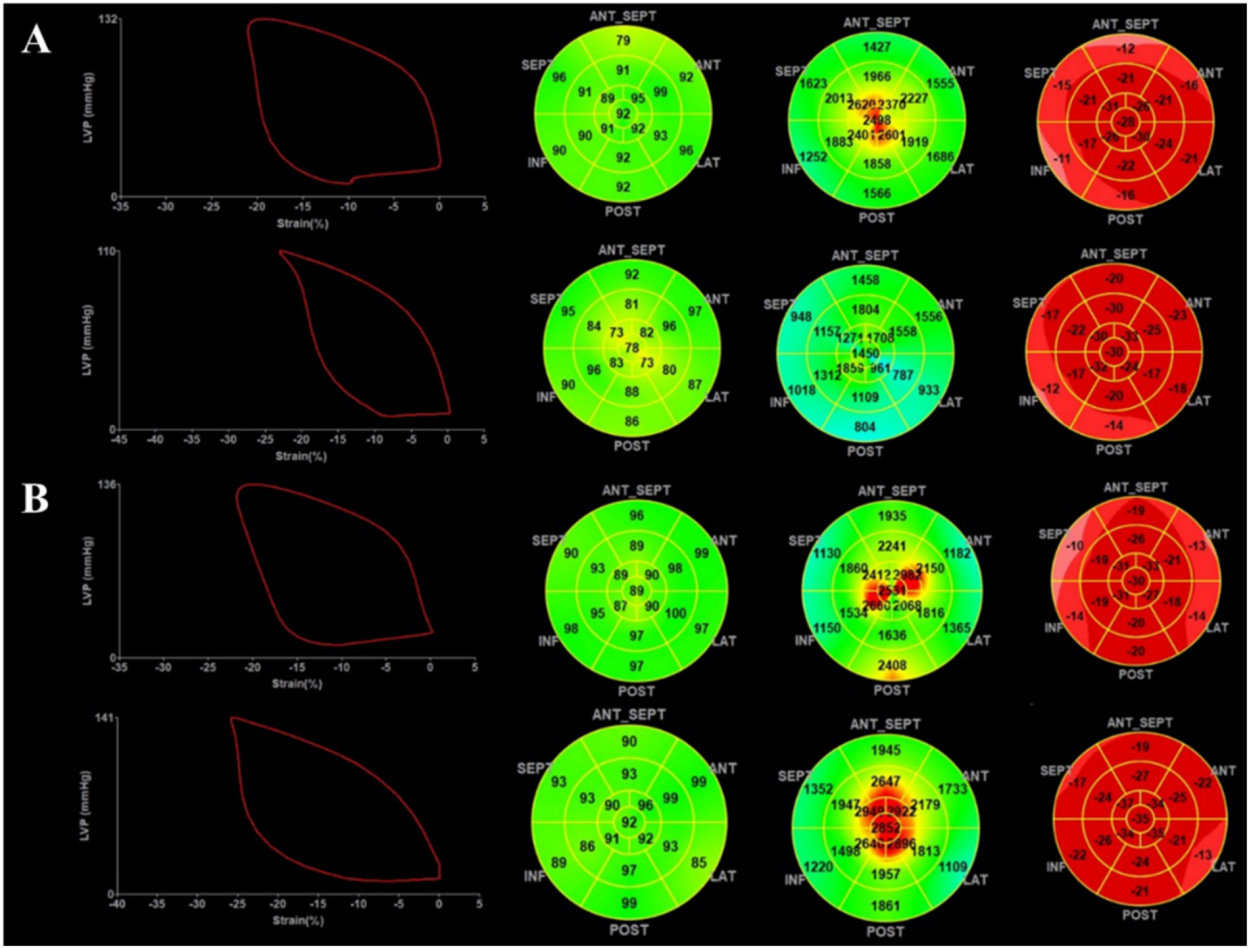
Demonstration of myocardial work during regadenoson stress. **(A)** Data from a patient with myocardial ischemia: the top shows myocardial work before stress, and the bottom shows myocardial work after stress. **(B)** Data from a patient without myocardial ischemia: the top shows myocardial work before stress, and the bottom shows myocardial work after stress.

In both groups, compared with the baseline, the heart rate (HR), SBP, DBP, LADV, LVEF, GLS, and GWW increased after regadenoson stress, while GWE decreased after stress. However, the responses of GWI and GCW to stress were different in the two groups. GWI and GCW tend to increase upon stress in CFVR > 2.5, while they tend to decrease significantly or with preserved efficiency in CFVR < 2.5 (Figure 2).

#### 3.3.2 Comparison between the CFVR < 2.5 and CFVR ≥ 2.5 group

The ΔSBP and ΔLVEF were lower in the CFVR < 2.5 group. The patients with myocardial ischemia had higher LAD velocity at baseline. All the baseline MW parameters were not statistically different in the two groups. The ΔGWI, peak GCW, and ΔGCW were lower in the CFVR < 2.5 group, as shown in Table 3. The GWI, GWW, and GWE at the peak were not significantly different in the two groups, as shown in Table 3.

**Table 3:** Comparison of stress echocardiography in the two groups.

Variable	Total (*n* = 115)	CFVR ≥ 2.5	CFVR < 2.5	*P*
		(*n* = 67)	(*n* = 48)	
CFVR, M (Q₁, Q₃)	2.75 (2.23, 3.15)	3.07 (2.84, 3.58)	2.12 (1.94, 2.32)	**<0** **.** **001**
Base HR/bpm, Mean ± SD	71.27 ± 10.83	70.22 ± 10.42	72.73 ± 11.32	0.223
Peak HR/bpm, M (Q₁, Q₃)	92 (85, 103)^a^	89 (83.5, 102)^a^	94 (87, 103.5)^a^	0.191
ΔHR/%, Mean ± SD	31.47 ± 15.97	31.98 ± 17.47	30.76 ± 13.75	0.687
Base SBP/mmHg, Mean ± SD	129.97 ± 12.26	128.45 ± 11.63	132.08 ± 12.91	0.117
Peak SBP/mmHg, M (Q₁, Q₃)	123 (116, 136)^a^	124.00 (119, 136)^a^	121.5 (114, 136.25)^a^	0.28
ΔSBP/mmHg, Mean ± SD	−3.31 ± 7.92	−1.27 ± 7.54	−6.15 ± 7.62	**<0** **.** **001**
Base DBP/mmHg, M (Q₁, Q₃)	80 (74, 87.5)	80 (74, 86)	79 (73.75, 89.5)	0.952
Peak DBP/mmHg, Mean ± SD	75.82 ± 11.85^a^	76.72 ± 12.28^a^	74.56 ± 11.23^a^	0.339
ΔDBP/%, M (Q₁, Q₃)	−5.71 (−11.76, 0.65)	−4.65 (−10, 1.35)	−9.5 (−12.25, −2.49)	0.06
Base WMSI, M (Q₁, Q₃)	1 (1,1.2)	1 (1,1.12)	1 (1,1.25)	0.58
Peak WMSI, M (Q₁, Q₃)	1 (1,1.3)	1 (1,1.21)	1 (1,1.28)	0.34
Base LADV/m/s, Mean ± SD	0.25 ± 0.09	0.22 ± 0.06	0.29 ± 0.1	**<0** **.** **001**
Peak LADV/m/s, M (Q₁, Q₃)	0.64 (0.52, 0.76)^a^	0.64 (0.55, 0.76)^a^	0.6 (0.47, 0.74)^a^	0.052
Base LVEF/%, Mean ± SD	65.09 ± 6.25	64.72 ± 5.92	65.61 ± 6.71	0.45
Peak LVEF/%, Mean ± SD	69.62 ± 6.55^a^	70.23 ± 6.39^a^	68.79 ± 6.74^a^	0.25
ΔLVEF/%, Mean ± SD	7.29 ± 8.63	8.79 ± 8.54	5.22 ± 8.4	**0** **.** **029**
Base GLS/%, Mean ± SD	−19.84 ± 3.27	−19.86 ± 3.08	−19.81 ± 3.56	0.928
Peak GLS/%, M (Q₁, Q₃)	−22.5 (−24.05 −20)^a^	−22.90 (−24.15 −21.25)^a^	−21.70 (−24 −19.5)^a^	0.138
ΔGLS/%, M (Q₁, Q₃)	10.96 (4.55, 19.25)	12.65 (4.82, 21.66)	9.38 (2.17, 18.06)	0.088
Base GWI/mmHg%, Mean ± SD	1,951.94 ± 396.37	1,897.81 ± 340.35	2,027.5 ± 456.51	0.1
Peak GWI/mmHg%, Mean ± SD	1,964.87 ± 453.97^a^	1,999.85 ± 418.05^a^	1,916.77 ± 499.72^a^	0.337
ΔGWI/%, Mean ± SD	0.99 ± 15.7	5.79 ± 16.15	−5.61 ± 12.45	**<0** **.** **001**
Base GCW/mmHg%, Mean ± SD	2,359.83 ± 426.95	2,317.12 ± 414.12	2,419.44 ± 441.68	0.206
Peak GCW/mmHg%, Mean ± SD	2,487.42 ± 473.19^a^	2,573.52 ± 437.3^a^	2,369.04 ± 499.14	**0** **.** **022**
ΔGCW/%, Mean ± SD	5.81 ± 14.12	11.60 ± 13.66	−2.15 ± 10.47	**<0** **.** **001**
Base GWW/mmHg%, M (Q₁, Q₃)	109 (67, 159)	119.00 (70, 167)	97.00 (66.75, 152.25)	0.335
Peak GWW/mmHg%, M (Q₁, Q₃)	143.5 (82.5, 222)^a^	150.5 (87, 245.5)^a^	133.5 (73.75,202.5)^a^	0.238
ΔGWW/%, M (Q₁, Q₃)	28.18 (−24.9, 100)	30.06 (−33.07, 101.4)	25.90(−16.35, 82.11)	0.979
Base GWE/mmHg%, M (Q₁, Q₃)	95 (93, 96)	95 (93, 96)	95 (94, 96)	0.633
Peak GWE/mmHg%, M (Q₁, Q₃)	94 (91, 96)^a^	94 (90.25, 96)^a^	93.50 (91.75, 96)^a^	0.809
ΔGWE/%, M (Q₁, Q₃)	−1.05 (−3.22, 1.04)	−0.51 (−4.19, 1.04)	−1.06 (−3.13, 0.26)	0.965

^a^ Statistically different between baseline and peak status.

CFVR, coronary flow velocity reserve; SBP, systolic blood pressure; DBP, diastolic blood pressure; LADV, velocity of left anterior descending artery; HR, heart rate; LVEF, left ventricular ejection fraction; GLS, global longitudinal strain; GWI, global work index; GCW, global constructive work; GWW, global waste work; GWE, global work efficiency.

The bold values mean *P* < 0.05.

### 3.4 Predictors of myocardial ischemia and modeling

We assessed multicollinearity by collinearity diagnosis (tolerance < 0.1, variance inflation factor >10). Firstly, univariate logistic regression was performed. The parameters with *P* < 0.1 were selected for multivariate logistic regression. The diagnostic value of the parameters was evaluated by receiver operating characteristic (ROC) curves, and the area under the curve (AUC) was calculated (Supplementary Table S1). ΔGCW was the single index with the highest diagnostic value (Supplementary Table S1). Age, Hb, △SBP, △LVEF, △GWI, Peak GCW, and △GCW were all included in the multivariate logistic regression. Hb (OR = 0.971, *P* = 0.008) and △GCW (OR = 0.894, *P* = 0.002) were independent predictors of CFVR abnormality after adjusting for confounders (Table 4).

**Table 4:** Logistic regression of the CFVR < 2.5 predictor.

Parameters	Univariate logistic regression		Multivariate logistic regression	
	OR (95% CI)	*P*	OR (95% CI)	*P*
Age	1.058 (1.010, 1.101)	**0**.**017**	1.018 (0.952, 1.088)	0.608
Hb	0.977 (0.956, 0.998)	**0**.**033**	0.971 (0.949, 0.992)	**0**.**008**
LVESV	0.966 (0.924, 1.009)	0.120		
ΔSBP	0.918 (0.871, 0.968)	**0**.**002**	0.978 (0.890, 1.075)	0.978
ΔLVEF	0.950 (0.907, 0.996)	**0**.**032**	0.964 (0.908, 1.024)	0.239
ΔGWI	0.947 (0.919, 0.976)	**0**.**001**	0.984 (0.939, 1.031)	0.507
Peak GCW	0.999 (0.998, 1.000)	**0**.**026**	1.000 (0.998, 1.001)	0.651
ΔGCW	0.902 (0.863, 0.944)	**0**.**001**	0.894 (0.833, 0.959)	**0**.**002**

The bold values mean *P* < 0.05.

**Figure 3:**
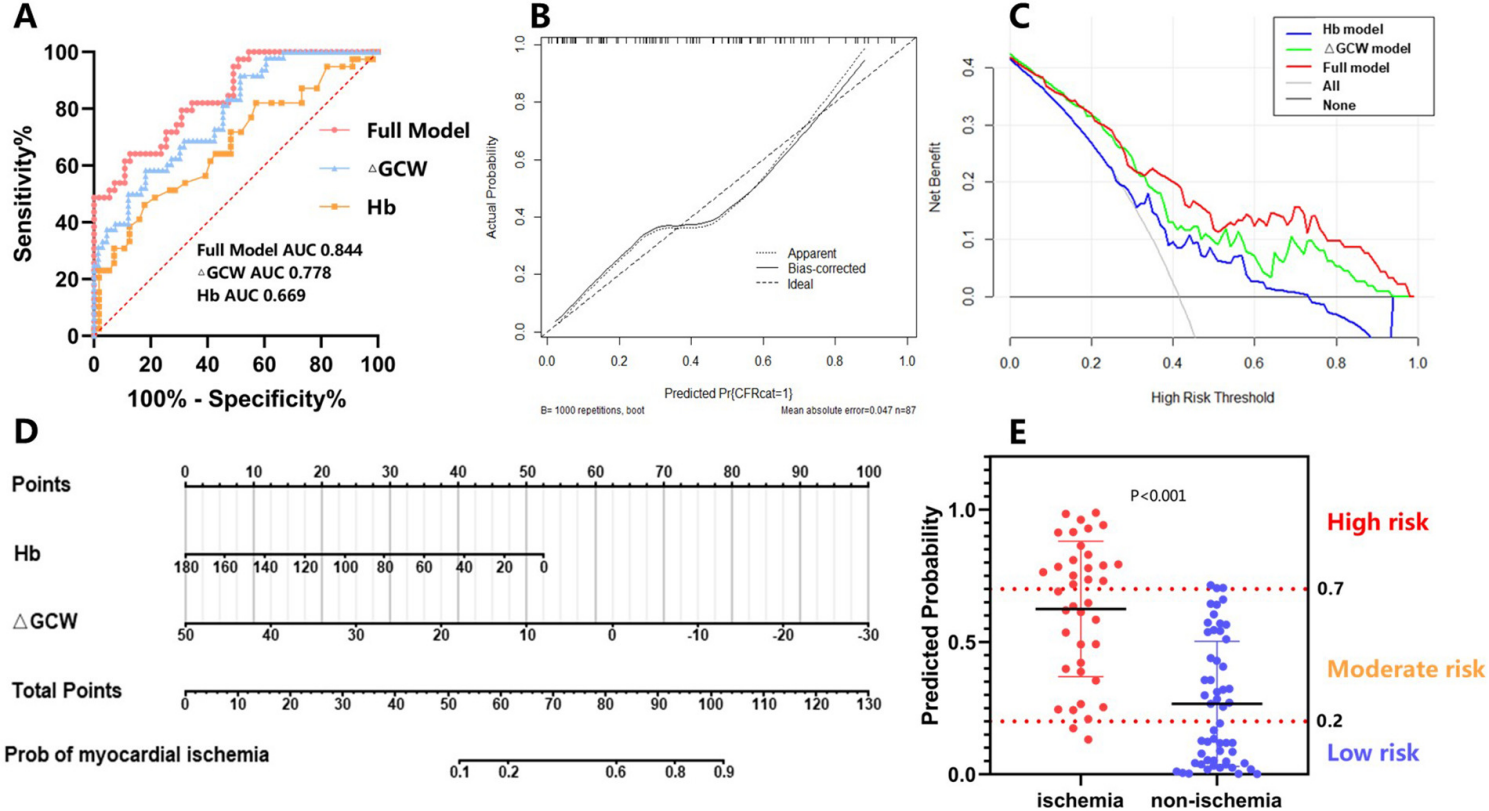
Evaluation of the model. **(A)** ROC curve analysis; **(B)** calibration analysis; **(C)** decision curve analysis; **(D)** nomograph; **(E)** risk stratification of the model.

We then developed a full model integrating Hb and ΔGCW. The ROC curve of the model had an AUC of 0.844, and ΔGCW contributed most to the discrimination of myocardial ischemia (Table 5, Figure 3A, Supplementary Table S1). ΔGCW was moderately related to CFVR (rho = 0.467, *P* < 0.001) (Supplementary Figure S1). The calibration ability of the model was evaluated by the Hosmer–Lemeshow goodness of fit (*χ*^2^ = 4.7337, *P* = 0.785) and calibration curve (Figure 3B). The decision curve analysis reflects the benefits of the full model compared with a single indicator for the identification of high-risk populations and further clinical management (Figure 3C). The CFVR < 2.5 probability developed by the logistic regression was expressed as follows: probability of CFVR < 2.5 = $\frac{1}{\left\{1+exp[-Logit(P)]\right\}}$, Logit(*P*) = 3.972 − 0.03 × Hb − 0.134 × ΔGCW. A personal myocardial ischemia could be conveniently calculated using nomography (Figure 3D). The different sensitivity and specificity of the model at different cutoff points were displayed in Supplementary Table S2. We defined the probability of 20% and 70% as the cutoff value of low, medium, and high risk. Among 22 patients classified into the high-risk group, 19 patients (86.36%) were proven to have myocardial ischemia. However, in 30 patients with a probability of <20%, only 2 patients (6.67%) had myocardial ischemia (Figure 3E).

**Table 5:** The performance of ΔGCW, Hb, and the new model combining ΔGCW and Hb for detecting myocardial ischemia.

Model variables	Discrimination		Reclassification				Goodness of fit
	AUC (95% CI)	*P*	NRI (95% CI)	*P*	IDI (95% CI)	*P*	AIC
Hb	0.668 (0.564, 0.762)	/	/	/	/	/	125.27
ΔGCW (%, vs. Hb)	0.777 (0.690, 0.850)	0.062	0.625 (0.349,0.902)	<0.001	0.187 (0.072,0.302)	0.001	130.41
ΔGCW + Hb (vs. ΔGCW)	0.844 (0.755, 0.911)	0.153	0.162 (−0.024, 0.346)	0.087	0.063 (0.007, 0.119)	0.028	97.55
ΔGCW + Hb (vs. Hb)	0.844 (0.755, 0.911)	0.004	0.787 (0.532, 1.042)	<0.001	0.250 (0.153,0.346)	<0.001	97.55

Finally, the predictive ability of the model was validated in a group of 62 patients. In this cohort, 31 patients (50%) had CFVR < 2.5. Of these 62 patients, 20 patients were defined as low risk, 16 (80%) of whom had true-negative diagnoses. In contrast, 10 patients were classified as high risk and were then totally confirmed as CFVR < 2.5 (Supplementary Figures S2A,B). The model showed good discrimination between patients with high risk and low risk of ischemia (AUC = 0.82) in the validation cohort (Supplementary Figure 2C).

### 3.5 Reproducibility

The reproducibility of MW was tested in 20 patients. Excellent intra-observer and inter-observer variabilities were observed in the measurement of MW parameters, which were demonstrated by intra-class correlation (ICC) (Supplementary Table S3) and Bland–Altman plots (Supplementary Figure S3).

## 4 Discussion

In this study, we analyzed the response of MW to regadenoson in CCS patients and subsequently, the feasibility of MW for predicting ischemia in CCS. We found that: (1) compared to the baseline status, GLS absolute value and GWW increased significantly by stress, and GWE decreased after stress. GWI and GCW would increase by stress in the CFVR ≥ 2.5 group but tend to decrease significantly or with preserved efficiency in the CFVR < 2.5 group. (2) After adjusting for confounding factors, ΔGCW and Hb are independent correlation factors for myocardial ischemia in CCS. (3) The novel full model integrating ΔGCW and Hb could be used in the estimation and risk stratification of ischemia. Non-invasive identification of high-risk patients has an important role in reducing unnecessary invasive coronary investigation and excessive revascularization.

### 4.1 MW outperforms GLS and RWMA in stress echocardiography for CCS

In the present study, general myocardial mechanical indices such as RWMA and GLS at rest and stress were not statistically significant between the two groups (19–22). Peak GLS only achieved an AUC of 0.581 (0.473–0.690) to predict CFVR abnormality, while ΔGCW achieved 0.777 (0.694–0.861) (Supplementary Table S1). MW is superior in the following aspects: Firstly, LV PSL is measured during the whole cardiac cycle, reflecting the energy utilization throughout the cardiac cycle (15). The use of multiple MW indices allows for quantitative evaluation of positive and negative output (23). Secondly, MW could overcome the afterload-dependent limitation of strain (24–26). Our study further demonstrates that the reserve of MW (ΔGCW) is a more accurate predictor of myocardial ischemia in CCS the absolute values of MW. In the ischemia group, the vasodilator would induce horizontal and vertical steel in the blood supply, resulting in uncoordinated local myocardial motion, impaired cardiac output. The GCW has precisely quantified the energy consumed by the myocardium that effectively contributes to cardiac output. Our study is consistent with the studies of Guo et al. (19) and Leitman et al. (27); GCW was sensitive to functional myocardial ischemia. But it is not consistent with Liu et al.'s (22) study that peak GLS differs in the coronary microvascular disease (CMD) group and the non-CMD group. It is possible that Liu et al.'s study was established in angina with non-obstructive coronary arteries rather than in heterogenous CCS. Consequently, the utilization of MW reserve in the diagnosis of myocardial ischemia may circumvent the intricacies and heterogeneities of CCS, offering a comparatively objective indicator of how diverse subtypes of CCS respond to stressors.

### 4.2 Multivariate diagnostic model in predicting myocardial ischemia

Moreover, we conduct a novel diagnostic model to predict myocardial ischemia in CCS, which is currently lacking. In the multivariate diagnostic model, we include ΔGCW and Hb to increase the diagnostic value from 0.777 to 0.844. Our study demonstrated that Hb is an independent risk factor for myocardial ischemia (OR = 0.971, 95% CI: 0.949–0.992), which is consistent with the ARIC cohort study. The ARIC study may be the first to suggest that anemia is an independent risk factor for ischemia-related cardiovascular outcomes in the general population (HR = 1.41, 95% CI: 1.01–1.95) (28). Numerous studies have also shown that anemia is associated with poor outcomes in patients with cardiovascular disease due to chronic inflammation, inhibition of the renin–angiotensin–aldosterone system, and renal dysfunction (29–31). The degree of anemia is therefore associated with myocardial ischemia.

A combination of clinical data and stress MW indices in a multivariate model might rectify the overlap of a single factor between the two groups. A further invasive investigation into coronary physiology could be more costly and technically challenging. With a probability calculator in this study, the probability of myocardial ischemia is very low if the probability is below 20%. Of the 30 participants in this study with a probability below 20%, only 2 had myocardial ischemia. These patients could be free from further invasive coronary physiology investigation. Those with a probability of >70% were classified into the high-risk category. The probability of myocardial ischemia is relatively high among 22 patients at high risk. Only three did not have myocardial ischemia. Tight control of lipid levels, the use of anti-angina therapy, and outpatient follow-up are essential if additional testing is not preferred. Additional testing is needed for those with moderate-risk (probability 20%–70%). The model was also applied in the validation cohort, which also showed good discrimination. The algorithm provides a framework that can be used to determine identified probability in the diagnosis of myocardial ischemia, based on a clinical and an echocardiographic parameter, rather than a binary category (present or absent).

### 4.3 Potential clinical implication

Reserve of MW could help us infer the probability of CFVR abnormality in vasodilator SE. Although this study was based on vasodilator stress, it also suggests that myocardial work reserve might be used to predict CFVR in situations with low CFVR success rates. The probability of myocardial ischemia in CCS could be calculated through the nomogram. The calculated likelihoods can assist clinicians in making clinical decisions.

### 4.4 Limitation

There are several potential limitations of the study. Firstly, this study was conducted at a single center with a small sample size, which may lead to statistical error. Further large-scale and multicenter studies need to verify the preliminary results. Secondly, the definition of myocardial ischemia was a CFVR abnormality. However, we merely measured CFVR in LAD. Myocardial ischemia in other coronary territories may be misdiagnosed. However, a study has shown that the LAD supplies approximately half of the myocardium, and ischemia in the region of LAD is strongly associated with prognosis (8). In the following study, we will validate the relation between MW and myocardial ischemia by SPECT or PET. Thirdly, only the response of MW to regadenoson was studied, and it remains unclear how MW in CCS changes under other stress modalities.

## 5 Conclusion

The incorporation of Hb and ΔGCW into the novel prediction model offers incremental value in estimating the likelihood of myocardial ischemia. The reserve of MW demonstrates predictive efficacy in identifying early myocardial ischemia.

## Data Availability

The raw data supporting the conclusions of this article will be made available by the authors, without undue reservation.

## References

[1] Mozaffarian D Benjamin EJ Go AS Arnett DK Blaha MJ Cushman M Heart Disease and Stroke Statistics—2016 update. Circulation. (2016) 133(4):e38–360. 10.1161/CIR.0000000000000350 26673558

[2] Knuuti J Wijns W Saraste A Capodanno D Barbato E Funck-Brentano C 2019 ESC guidelines for the diagnosis and management of chronic coronary syndromes. Eur Heart J. (2020) 41(3):407–77. 10.1093/eurheartj/ehz425 31504439

[3] Tonino PAL Fearon WF De Bruyne B Oldroyd KG Leesar MA Ver Lee PN Angiographic versus functional severity of coronary artery stenoses in the FAME study fractional flow reserve versus angiography in multivessel evaluation. J Am Coll Cardiol. (2010) 55(25):2816–21. 10.1016/j.jacc.2009.11.096 20579537

[4] Zimmermann Frederik M. Ding Victoria Y. Pijls Nico H. J. Piroth Zsolt van Straten Albert H. M. Szekely Laszlo Davidavicius Giedrius 2023. Fractional flow reserve-guided PCI or coronary bypass surgery for 3-vessel coronary artery disease: 3-year follow-up of the FAME 3 trial. Circulation 148 (12): 950–58. 10.1161/CIRCULATIONAHA.123.065770 37602376

[5] Ciampi Q Zagatina A Cortigiani L Wierzbowska-Drabik K Kasprzak JD Haberka M Prognostic value of stress echocardiography assessed by the ABCDE protocol. Eur Heart J. (2021) 42(37):3869–78. 10.1093/eurheartj/ehab493 34449837 PMC8486488

[6] Budoff MJ Mayrhofer T Ferencik M Bittner D Lee KL Lu MT Prognostic value of coronary artery calcium in the PROMISE study (prospective multicenter imaging study for evaluation of chest pain). Circulation. (2017) 136(21):1993–2005. 10.1161/CIRCULATIONAHA.117.030578 28847895 PMC5698136

[7] Shaw LJ Berman DS Maron DJ John Mancini GB Hayes SW Hartigan PM Optimal medical therapy with or without percutaneous coronary intervention to reduce ischemic burden: results from the clinical outcomes utilizing revascularization and aggressive drug evaluation (COURAGE) trial nuclear substudy. Circulation. (2008) 117(10):1283–91. 10.1161/CIRCULATIONAHA.107.743963 18268144

[8] Picano E Pierard L Peteiro J Djordjevic-Dikic A Sade LE Cortigiani L The clinical use of stress echocardiography in chronic coronary syndromes and beyond coronary artery disease: a clinical consensus statement from the European Association of Cardiovascular Imaging of the ESC. Eur Heart J Cardiovasc Imaging. (2024) 25(2):e65–90. 10.1093/ehjci/jead250 37798126

[9] Dagianti A Penco M Agati L Sciomer S Dagianti A Rosanio S Stress echocardiography: comparison of exercise, dipyridamole and dobutamine in detecting and predicting the extent of coronary artery disease. J Am Coll Cardiol. (1995) 26(1):18–25. 10.1016/0735-1097(95)00121-f 7797748

[10] Wierzbowska-Drabik K Picano E Cortigiani L Kasprzak JD. Comparison of coronary flow reserve feasibility in different stress echocardiography protocols: dobutamine, dipyridamole, exercise, and rapid pacing. Pol Arch Int Med. (2021) 131(9):830–9. 10.20452/pamw.16035 34142788

[11] Gaibazzi N Ciampi Q Cortigiani L Wierzbowska-Drabik K Zagatina A Djordjevic-Dikic A Multiple phenotypes of chronic coronary syndromes identified by ABCDE stress echocardiography. J Am Soc Echocardiogr. (2023) 37(5):477–85. 10.1016/j.echo.2023.12.003 38092306

[12] Cortigiani L Urluescu M-L Coltelli M Carpeggiani C Bovenzi F Picano E. Apparent declining prognostic value of a negative stress echocardiography based on regional wall motion abnormalities in patients with normal resting left ventricular function due to the changing referral profile of the population under study. Circulation. (2019) 12(6):e008564. 10.1161/CIRCIMAGING.118.008564 31167561

[13] Russell K Eriksen M Aaberge L Wilhelmsen N Skulstad H Remme EW A novel clinical method for quantification of regional left ventricular pressure–strain loop area: a non-invasive Index of myocardial work. Eur Heart J. (2012) 33(6):724–33. 10.1093/eurheartj/ehs016 22315346 PMC3303715

[14] Roemer S Jaglan A Santos D Umland M Jain R Jamil Tajik A The utility of myocardial work in clinical practice. J Am Soc Echocardiogr. (2021) 34(8):807–18. 10.1016/j.echo.2021.04.013 33895250

[15] Edwards NFA Scalia GM Shiino K Sabapathy S Anderson B Chamberlain R Global myocardial work is superior to global longitudinal strain to predict significant coronary artery disease in patients with normal left ventricular function and wall motion. J Am Soc Echocardiogr. (2019a) 32(8):947–57. 10.1016/j.echo.2019.02.014 31043359

[16] Chen W Ni M Huang H Cong H Fu X Gao W Chinese expert consensus on the diagnosis and treatment of coronary microvascular diseases (2023 edition). MedComm. (2023) 4(6):e438. 10.1002/mco2.438 38116064 PMC10729292

[17] Mitchell C Rahko PS Blauwet LA Canaday B Finstuen JA Foster MC Guidelines for performing a comprehensive transthoracic echocardiographic examination in adults: recommendations from the American Society of Echocardiography. J Am Soc Echocardiogr. (2019) 32(1):1–64. 10.1016/j.echo.2018.06.004 30282592

[18] Pellikka PA Arruda-Olson A Chaudhry FA Chen MH Marshall JE Porter TR Guidelines for performance, interpretation, and application of stress echocardiography in ischemic heart disease: from the American Society of Echocardiography. J Am Soc Echocardiogr. (2020) 33(1):1–41.e8. 10.1016/j.echo.2019.07.001 31740370

[19] Guo Y Yang C Wang X Pei Z Zhu H Meng X Regional myocardial work measured by echocardiography for the detection of myocardial ischemic segments: a comparative study with invasive fractional flow reserve. Front Cardiovasc Med. (2022) 9:813710. 10.3389/fcvm.2022.813710 35369304 PMC8965858

[20] Boe E Russell K Eek C Eriksen M Remme EW Smiseth OA Non-invasive myocardial work Index identifies acute coronary occlusion in patients with non-ST-segment elevation-acute coronary syndrome. European Heart Journal. Cardiovascular Imaging. (2015) 16(11):1247–55. 10.1093/ehjci/jev078 25851329

[21] Clemmensen TS Eiskjær H Mikkelsen F Granstam S-O Flachskampf FA Sørensen J Left ventricular pressure-strain-derived myocardial work at rest and during exercise in patients with cardiac amyloidosis. J Am Soc Echocardiogr. (2020) 33(5):573–82. 10.1016/j.echo.2019.11.018 32061410

[22] Liu Q Li Q Wan X Xu M Pan J Zhang Y The value of myocardial work in the estimation of left ventricular systolic function in patients with coronary microvascular disease: a study based on adenosine stress echocardiography. Front Cardiovasc Med. (2023) 10:1119785. 10.3389/fcvm.2023.1119785 37113699 PMC10126338

[23] Moya A Buytaert D Penicka M Bartunek J Vanderheyden M. State-of-the-art: noninvasive assessment of left ventricular function through myocardial work. J Am Soc Echocardiogr. (2023) 36(10):1027–42. 10.1016/j.echo.2023.07.002 37437670

[24] Reant P Metras A Detaille D Reynaud A Diolez P Jaspard-Vinassa B Impact of afterload increase on left ventricular myocardial deformation indices. J Am Soc Echocardiogr. (2016) 29(12):1217–28. 10.1016/j.echo.2016.09.006 27751650

[25] Donal E Bergerot C Thibault H Ernande L Loufoua J Augeul L Influence of afterload on left ventricular radial and longitudinal systolic functions: a two-dimensional strain imaging study. Eur J Echocardiogr. (2009) 10(8):914–21. 10.1093/ejechocard/jep095 19666722

[26] Marzlin N Hays AG Peters M Kaminski A Roemer S O’Leary P Myocardial work in echocardiography. Circulation. Cardiovascular Imaging. (2023) 16(2):e014419. 10.1161/CIRCIMAGING.122.014419 36734221

[27] Leitman M Balboul Y Burgsdorf O Tyomkin V Fuchs S. Myocardial work index during normal dobutamine stress echocardiography. Sci Rep. (2022) 12:6813. 10.1038/s41598-022-10903-8 35473955 PMC9042838

[28] Sarnak MJ Tighiouart H Manjunath G MacLeod B Griffith J Salem D Anemia as a risk factor for cardiovascular disease in the Atherosclerosis Risk in Communities (ARIC) study. J Am Coll Cardiol. (2002) 40(1):27–33. 10.1016/s0735-1097(02)01938-1 12103252

[29] Sabatine MS Morrow DA Giugliano RP Burton PBJ Murphy SA McCabe CH Association of hemoglobin levels with clinical outcomes in acute coronary syndromes. Circulation. (2005) 111(16):2042–49. 10.1161/01.CIR.0000162477.70955.5F 15824203

[30] McClellan WM Dana Flanders W Langston RD Jurkovitz C Presley R. Anemia and renal insufficiency are independent risk factors for death among patients with congestive heart failure admitted to community hospitals: a population-based study. J Am Soc Nephrol. (2002) 13(7):1928–36. 10.1097/01.asn.0000018409.45834.fa 12089390

[31] Horwich TB Fonarow GC Hamilton MA MacLellan WR Borenstein J. Anemia is associated with worse symptoms, greater impairment in functional capacity and a significant increase in mortality in patients with advanced heart failure. J Am Coll Cardiol. (2002) 39(11):1780–86. 10.1016/s0735-1097(02)01854-5 12039491

