# Supplementary material for: Reserve of global constructive work for early diagnosis of myocardial ischemia and risk stratification in chronic coronary syndrome
Source: Front Cardiovasc Med. 2025 Aug 1;12:1598453. doi: 10.3389/fcvm.2025.1598453 (PMC12354653; doi:10.3389/fcvm.2025.1598453)
Supplement: Supplementary file 1 [file Table1.pdf]

**Supplementary table 1 ROC analysis of model and parameters**

| Parameters | AUC | 95%CI |
|---|---|---|
| Full model | 0.844 | 0.767-0.921 |
| ΔGCW | 0.777 | 0.694-0.861 |
| ΔGWI | 0.712 | 0.537-0.740 |
| Peak GCW | 0.621 | 0.618-0.805 |
| Peak GLS | 0.581 | 0.473-0.690 |
| ΔSBP | 0.669 | 0.570-0.769 |
| Age | 0.632 | 0.528-0.735 |
| ΔLVEF | 0.618 | 0.513-0.723 |

**Supplementary table 2 Sensitivity and specificity of CFVR<2.5 model at different cut-off value**

| Cut-off value | Sensitivity (95%CI) | Specificity (95%CI) |
|---|---|---|
| 0.20 | 94.87% (82.7%- 99.4%) | 50.91% (37.1%- 64.6%) |
| 0.40 | 74.36% (57.9%- 87.0%) | 70.91% (57.1%- 82.4%) |
| 0.60 | 61.54% (44.6%- 76.6%) | 89.09% (77.8%- 95.9%) |
| 0.70 | 48.72% (2.5%- 31.2%) | 100% (93.5%- 100.0%) |

**Supplementary table 3 Reproducibility of myocardial work**

| | intra-observer | | | inter-observer | | |
|---|---|---|---|---|---|---|
| | **ICC** | **95%CI** | **P** | **ICC** | **95%CI** | **P** |
| GWI | 0.962 | 0.908-0.985 | P<0.001 | 0.946 | 0.869-0.978 | P<0.001 |
| GCW | 0.943 | 0.861-0.977 | P<0.001 | 0.923 | 0.818-0.969 | P<0.001 |
| GWW | 0.728 | 0.432-0.883 | P<0.001 | 0.711 | 0.404-0.874 | P<0.001 |
| GWE | 0.798 | 0.558-0.915 | P<0.001 | 0.679 | 0.353-0.859 | P<0.001 |
| GLS | 0.745 | 0.461-0.891 | P<0.001 | 0.834 | 0.626-0.931 | P<0.001 |

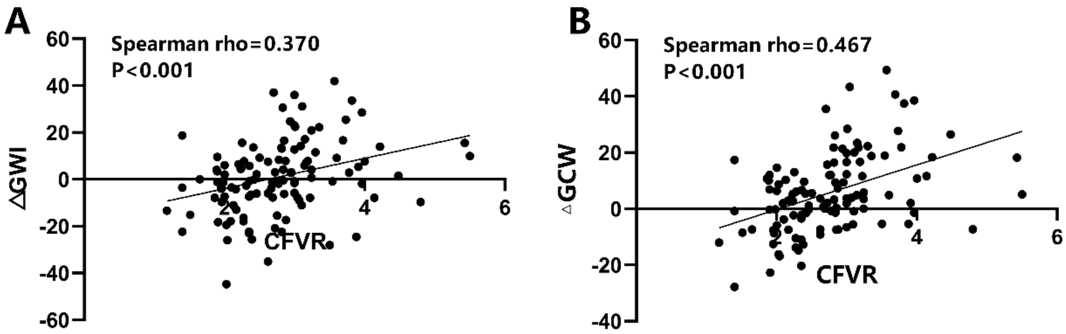


**Supplementary Figure 1. Correlation between △GWI, △GCW and CFVR**

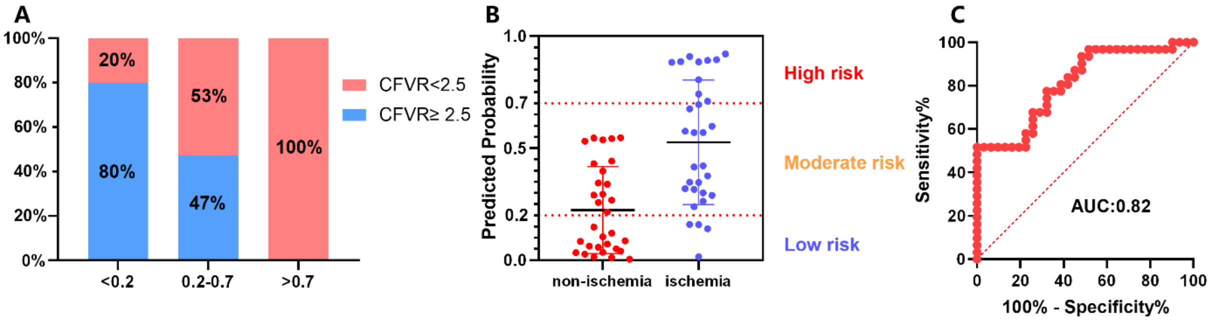


**Supplementary Figure 2. Discrimination value in the validation cohort**

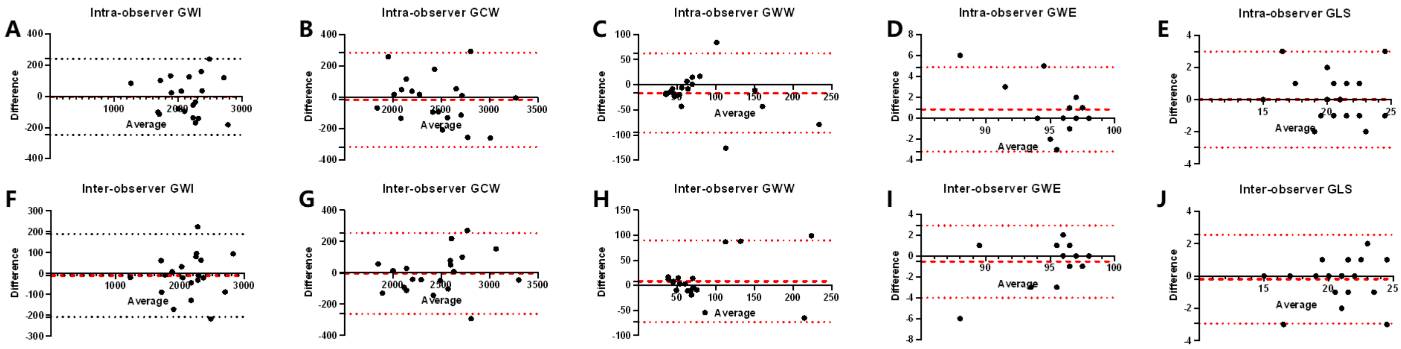


**Supplementary Figure 3. Bland-Altmann Plots of myocardial work indices.** Intra-observer GWI, GCW, GWW, GWE, GLS(A-E), inter-observer GWI, GCW, GWW, GWE, GLS (F-J) variability of myocardial work as shown in Bland-Altmann Plots.
